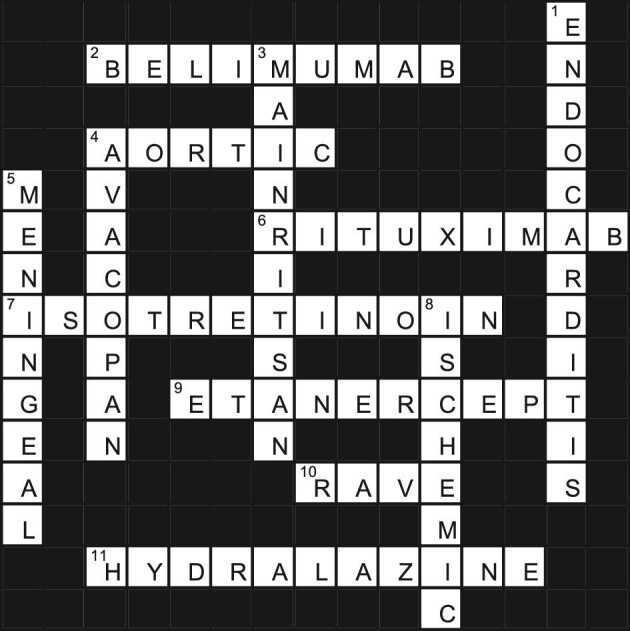# Podocyte puzzle: ANCA vasculitis

**DOI:** 10.1093/ckj/sfad278

**Published:** 2023-11-09

**Authors:** Kate Stevens, David Kipgen, Kenar D Jhaveri

**Affiliations:** Glasgow Renal and Transplant Unit, Queen Elizabeth University Hospital, Glasgow, UK; Department of Pathology, Queen Elizabeth University Hospital, Glasgow, UK; Glomerular Center at Northwell Health, Division of Kidney Diseases and Hypertension, Northwell Health, Great Neck, NY, USA

 

**Figure 1:**
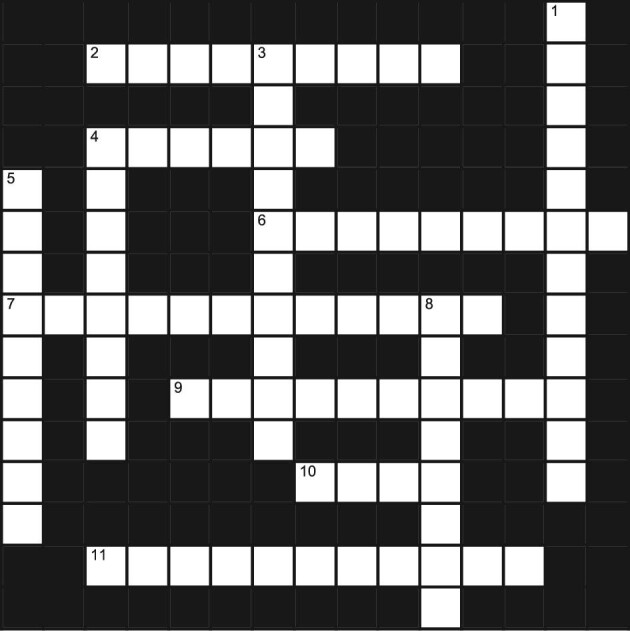


Across

2 This anti BAFF agent used in SLE and lupus nephritis is under investigation in treatment of ANCA vasculitis. (9)
4 A 44-year-old man with pansinusitis, otitis, mastoiditis, and chronic lymphocytic meningitis with positive pANCA and anti-MPO antibodies presents with a three-out-of-four diastolic murmur with echocardiogram showing septal hypertrophy leading to aortic valve closure. His cardiac disease is ____ regurgitation. (6)
6 This anti B cell agent is used in combination with cyclophosphamide as induction based on RITUXVAS protocol. (9)
7 A 19-year-old man with severe acne conglobata and ulcerated pyoderma gangrenosum on the face was prescribed this medication by a dermatologist. Two weeks later his internist notes 2.5 gm of proteinuria, hematuria, and positive c-ANCA. A kidney biopsy confirms pauci immune glomerular nephritis. (12)
9 A 50-year-old woman with progressive rheumatoid arthritis with erosive joint disease on methotrexate was prescribed this medication by her rheumatologist. At a follow-up visit 1 month later, she reports lethargy, upper respiratory tract symptoms, and a skin rash. She is found to have an elevated serum creatinine of 2.5 mg/dL, a eGFR of 22 ml/min/1.73m^2^, and a positive c-ANCA. Skin and kidney biopsies are performed, which diagnose small vessel vasculitis and necrotizing pauci immune crescentic glomerulonephritis, respectively. (10)
10 The ____ trial was a randomized, placebo-controlled, multicenter, noninferiority trial that compared induction therapy with rituximab (375 mg/m^2^ per week for four weeks) or oral cyclophosphamide (2 mg/kg per day) in close to 200 patients with ANCA vasculitis. (4)
11 This anti-hypertensive drug is known to cause ANCA vasculitis. (11)

Down

1 This infection is all heart when it comes to production of ANCA. (12)
3 The best data supporting the use of rituximab as maintenance therapy comes from the _____ trial that compared rituximab with azathioprine in 115 patients who had attained remission after initial therapy using cyclophosphamide plus glucocorticoids. (10)
4 Many clinicians are complimentary about this adjunctive agent used with standard induction therapy to limit the use of glucocorticoids. (8)
5 ______ disease is most associated with granulomatous inflammation of the central nervous system. (9)
8 The most common cardiac disease in ANCA vasculitis patients is ____ in nature. (8)

**Figure 1a:**
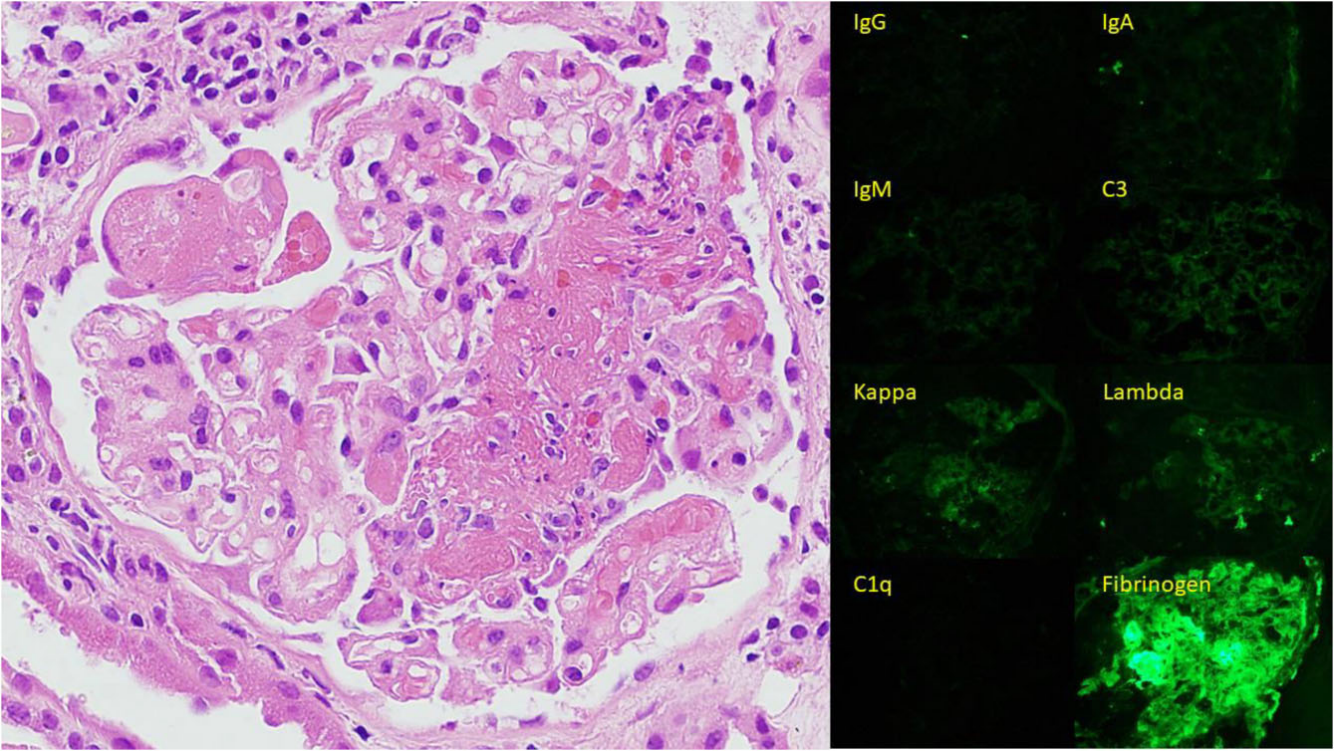
High power H + E image showing glomerulus with segmental fibrinoid necrosis and cellular crescent. Immunofluorescence images show fibrinogen in a glomerulus with fibrinoid necrosis. There is no significant deposition of immunoglobulins or complement (pauci-immune).

**Figure 1b:**